# Mean platelet volume and platelet counts in type 2 Diabetes: Mellitus on treatment and non-diabetic mellitus controls in Lagos, Nigeria

**DOI:** 10.11604/pamj.2014.18.42.3651

**Published:** 2014-05-12

**Authors:** Akinbami Akinsegun, Dada Akinola Olusola, John-Olabode Sarah, Oshinaike Olajumoke, Adediran Adewumi, Odesanya Majeed, Ogbera Anthonia, Uche Ebele, Okunoye Olaitan, Arogundade Olanrewaju, Aile Kingsley

**Affiliations:** 1 Department of Haematology and Blood Transfusion, Lagos State University, College of Medicine, Ikeja, Nigeria; 2 Department of Medicine, Lagos State University, College of Medicine, Ikeja, Nigeria; 3 Department of Haematology, Ben Carson School of Medicine, Babcock University, Ilisan-Remo, Ogun State, Nigeria; 4 Department of Haematology, Faculty of Clinical Sciences, College of Medicine, University of Lagos, Idiaraba, Nigeria; 5 Oak Hospitals, Ikorodu, Lagos, Nigeria; 6 Department of Medicine, University of PortHarcourt, River State, Nigeria; 7 Department of Haematology and Blood Transfusion, Lagos State University, Teaching Hospital, Ikeja, Nigeria

**Keywords:** Mean platelet volume, platelet count, Type 2 diabetics on treatment, Controls

## Abstract

**Introduction:**

The Mean platelet volume and platelet counts are indicators of thrombotic potentials, and risk factors for microvascular complications in diabetics. This study aimed to establish variations in platelet counts and mean platelet volume in type 2 diabetic patients on treatment and non-diabetic controls.

**Methods:**

This was an unmatched case-control study involving 200 participants consisting of 100 diabetics and 100 non-diabetic controls. Four and half milliliters of blood was collected from diabetics and non diabetic controls into EDTA anticoagulant tubes. Full blood count was performed using the Sysmex KN-21N, (manufactured by Sysmex corporation Kobe, Japan) a three- part auto analyzer able to run 19 parameters per sample including platelet counts and mean platelet volume.

**Results:**

The mean fasting blood sugar for the diabetics was 147.85±72.54 mg/dl and the controls 95.20±30.10 mg/dl. The mean platelet count for the diabetics was 235.29±76.81*10^9^/L and controls, 211.32±66.44*10^9^/L. The mean platelet volume, for the diabetics was 8.69±0.67 fl and the controls, 8.91±0.80 fl. There was a statistically significant difference in platelet counts of diabetics and healthy controls p =0.038 while none existed between the mean platelet volume in diabetics and healthy controls p = 0.593.

**Conclusion:**

This study revealed a higher mean platelet count for diabetics on treatment than for non diabetic controls while mean platelet volume was lower in cases than controls. However, both parameters in diabetics on treatment were within the normal reference range for healthy individuals.

## Introduction

Type 2 Diabetes mellitus (DM) accounts for 80% of all DM [1]. An interaction between environmental and genetic factors is responsible for the development of type 2 DM [1]. DM is characterized by enhanced platelets activation and coagulation proteins and reduced fibrinolytic activity [2]. This pro thrombotic state precede the development of cardiovascular and atherosclerotic complications associated with DM [3]. Type 2 diabetes mellitus patients have two-four folds increase in risk of atherosclerosis [4]. Luscher et al [5] also documented an increased risk of coronary artery disease and cerebrovascular disease as a result of accelerated atherosclerosis in DM. In type 2 DM, platelet function is of pathophysiological importance in atherothrombosis [6] Several authors have documented that increased morbidity and mortality in type 2 DM are associated with macro vascular (cardiovascular diseases, stroke, and peripheral arterial disease) and micro vascular (nephropathy, neuropathy and retinopathy) complications due to platelet dysfunction [7–9]. Also, an increased platelet counts and activity have been reported in diabetics as demonstrated by increases in GPs IIb/IIIa, 1b-IX, and 1a/IIa [10], CD62 and CD63 [11] Mean Platelet Volume (MPV), the average volume of platelets, a parameter in full blood count measures platelet size distribution, and is not influenced by glycaemic control [12]. An increased MPV has been associated with high incidence of proliferative diabetic retinopathy [13] and myocardial infarction [14]. An activated megakaryocyte-platelet system in diabetes mellitus has been reported to be responsible for larger than normal platelets circulating in DM patients [15]. Platelet count and MPV are simple, effective and cheap tests that may be used to predict angiopathy in type 2 DM. Elevated MPV has been documented to predict bad outcome for acute ischaemic cerebrovascular events independent of other clinical parameters [16]. This study aimed to establish variations in platelet counts and mean platelet volume in type 2 diabetic patients on treatment and non-diabetic controls. To the best of our knowledge this study is novel in our environment and will serve as a foundation for other researchers in this field.

## Methods

**Study Population:** This was a case-control study of 100 type 2 diabetic patients on treatment attending the diabetic clinic of Lagos State University Teaching Hospital (LASUTH) and 100 non diabetic controls consisting of medical students, nurses and doctors in the Institution. During the study period between June 2013 to September 2013 all patients who gave informed consent and satisfied the study inclusion criteria were recruited into the study. They were asked to fill structured questionnaires including demographic information, height, weight, last fasting blood sugar, blood pressure, drug history, and family history of diabetes. All the cases were on oral hypoglycaemic and antiplatelet drugs like clopridogel and vasoprin tablets, some were on antihypertensive and lipid lowering drugs. Information on family history of diabetes was also obtained from the controls and they were subjected to fasting blood sugar before enlistment.

**Ethics:** The research was approved by the Ethics Review Committee of LASUTH.

**Inclusion Criteria for the cases:** All non-insulin dependent diabetes mellitus patients on treatment attending the diabetes clinic.

**Exclusion Criteria for the cases:** Non-diabetic patients and insulin-dependent diabetes mellitus patients.

**Inclusion Criteria for the controls:** All consenting non-diabetics adults.

**Exclusion Criteria for the controls:** Diabetics adults on oral hypoglycaemic drugs.

**Sample Collection:** Blood specimen was withdrawn with minimal stasis from the ante-cubital vein using a dry sterile disposable syringe and needle. Four and half milliliters of blood was dispensed into EDTA anticoagulant tubes. The specimens were labeled with subject's age, sex and identification number. The EDTA samples were kept at room temperature until processed within 4hours of collection.

**Laboratory Analysis:** Full blood count was performed using the Sysmex KN-21N, (manufactured by Sysmex corporation Kobe, Japan) a three- part auto analyzer able to run 19 parameters per sample including haemoglobin concentration, packed cell volume, red blood cell concentration, mean corpuscular haemoglobin, mean cell volume, mean corpuscular haemoglobin concentration, white blood cells and platelet count and mean platelet volume. Standardization, calibration of instrument and processing of samples were done according to manufacturer's instructions.

**Procedure:** Well mixed blood sample was aspirated, by letting the equipment sampling probe into the blood sample and then pressing the start button. Approx. 20ul of blood was aspirated by the auto analyzer. Result of analysis is displayed after about 30secs. A printout copy of result is released on the thermal printing paper.

**Statistical Analysis:** Data were analyzed using SPSS version 16.0 (Statistical Package for Social Sciences, Inc., Chicago, Ill). The continuous variables were given as means ± standard deviation (SD). The Pearson chi squared test was used to test for association between discrete variables. P value was considered to be statistically significant when < 0.05.

## Results

A total of 200 participants were enrolled into the study consisting of 100 diabetics and 100 non-diabetic controls. The mean age of the controls was 32.38±66.44 years with a minimum 17 years and a maximum of 70 years. The mean age of diabetics was 62.35 ± 9.84 years, the minimum was 34 years and the maximum 90 years. The overall female: male ratio was 68%:32%, while the gender distribution in diabetics was 73%:27%, for the controls it was 63%:37% respectively (Table 1). The mean body mass index and fasting blood sugar of the diabetics were 32.10±4.85 kg/m^2^ and 147.85±72.54 mg/dl respectively. For the controls they were 25±5.23 kg/m2 and 95.20±30.10 mg/dl respectively. Amongst the diabetics, a total of 45 of 100 (45%) gave a positive family history of diabetes while 55% had no family history of diabetics. Only 5% of the controls gave a positive family history of diabetics. The mean duration of diabetics in the cases was 8.81±7.06 years. The overall mean platelet count was 223.49 ± 72.71 *109/L, for the diabetics 235.29±76.81*109/L and controls, 211.32±66.44*109/L. The overall mean platelet volume was 8.8±0.74fl, for the diabetics 8.69±0.67 fl and the non diabetic controls 8.91±0.80 fl (Table 2). There was a statistically significant difference in platelet count of diabetics and healthy controls platelet counts. p =0.038 while there was no statistically significant difference between the mean platelet volume in diabetics and healthy controls p = 0.593. Independent groups t-test for platelet counts and mean platelet volume showed t-statistics of 2.36 and 2.108 while the two tail probabilities of 0.0192 and 0.0363 respectively were obtained. Among the diabetics, a positive statistical Pearson's correlation was seen between MPV and fasting blood sugar (r = 0.04, p <0.001), body mass index (r =0.142) and duration of diabetics (r =0.045). While a negative correlation was seen between platelet count and fasting blood sugar, (r = -0.059), duration of diabetics (r = -0.027) but a positive correlation between platelet count and body mass index ( r = 0.002) Correlating platelet count with mean platelet volume using Pearson's test in both the diabetic patients and non diabetic controls showed statistically significant levels of 0.000 in both groups.(r = -0.485 p < 0.05;and r = -0.403 p < 0.05 respectively).

**Table 1:** Sociodemographic data of participants

Parameters	Diabetics (n = 100)	Non diabetic Controls(n = 100)
**Mean Age**		
Gender	62.35±9.84	32.38±66.44
Female	73	63
Male		
Educational Status	27	37
No Education	22	Nil
Primary	16	Nil
Secondary	24	Nil
Tertiary	38	100
Mean BMI	32.10±4.85	25±5.23

Abbreviations: BMI = Body mass index

**Table 2:** Mean platelet counts, MPV and FBS

Parameters	Diabetics	Non diabetic Controls
Mean Platelet counts	235.29±76.81	211.32±66.44 (p = 0.038)
Mean MPV	8.69±0.67	8.91±0.80 (p = 0.593)
Mean FBS	147.85±72.54	95.20±30.10

Abbreviations: MPV= mean platelet volume, FBS= fasting blood sugar

## Discussion

The MPV and platelet counts are indicators of thrombotic potential, and risk factors for microvascular complications in diabetics [17–19]. MPV is an indicator of the average size and activity of platelets, with a higher MPV value indicating a larger average platelet size. Larger platelets synthesize more thromboxane A2, are able to aggregate better, and are able to secrete more serotonin and β-thromboglobulin than smaller platelets [20–22]. Our study revealed a higher mean platelet count for diabetics than for the controls. This was in consonance with the findings by Thomas et al. [23], Zuberi et al [18], and Demirtunc et al. [24]. It is however in contrast with the findings of a study conducted by Hekimsoy et al. [17]. This suggests that the platelet count is the net result of the interplay of platelet survival and platelet production rate. The overall mean platelet volume was lower for the diabetics than for the non-diabetic controls. This was in contrast with the findings by Shah et al [25], Ates et al. [21], Hekimsoy et al [17], Demirtunc et.al [24], Zuberi et al. [18], Jindal et al. [26]. Papanas et al [27], and Thomas et al. [23]. However, this difference was not significant when subjected to statistical testing. (p = 0.593). The discordant result in our study may be accounted for by the fact that the majority of diabetics utilized for this study had been on treatment and in particular antiplatelet medications like clopidogrel and vasoprin for varying durations. This could impact the outcome of our result and a possible limitation of the study. Clopidogrel acts by irreversibly inhibiting the P2Y12 ADP receptor subtype on the platelet cell membrane, thereby preventing platelet activation and cross-linking by fibrin [28]. Activated platelets are larger, and prevention of platelet activation may prevent an increase in average platelet size and the mean MPV. This suggests that antiplatelet medication may reduce the thrombotic potential without causing a reduction in the absolute platelet count. We however cannot postulate that clopidogrel alone accounts for this effect. More studies would be required to explicitly describe these characteristics and define a time and dose-response relationship. The mean glycemic control of the diabetics utilized for our study was suboptimal (147.85±72.54 mg/dl). This may be accounted for by poor compliance to dietary modifications, lifestyle modifications and medications. We found a significant positive relationship between the MPV and glycemic control, as measured with the fasting blood sugar. A significant positive relationship was also seen in studies conducted by Shah et al, [25] who utilised HBA1c and fasting blood sugar levels, and Thomas et al and Demirtunc et al, who utilised the HBA1c levels of the patients [23, 24] This strongly indicates that achieving good glycemic control may limit platelet activation, and delay the onset or progression of microvascular complications in diabetics.

Our finding of a significant positive relationship between the MPV and the duration of diabetes gives credence to the fact that the risk of microvascular complications increases with the duration of diabetes. Discordant results were however found in studies conducted by Thomas et al and Hekimsoy et al. [23, 17]. This suggests that other factors may account for the thrombotic potential of diabetics with time [29]. More studies would be required to clarify this relationship. We also found a positive relationship between the MPV and BMI, unlike Thomas et al and Hekimsoy et al, who found no association [23, 17]. A falsely low platelet counts (pseudo-thrombocytopenia) may be due to misidentification of giant platelets as red cells by the automated platelet counts, other causes are EDTA-induced platelet clumping and satellitism [30]. A falsely high count may be due to markedly microcytic or fragemented red cells due to bacteria or fungi infection [31]. It is noteworthy that the mean platelet concentrations and mean platelet volume of both diabetics on treatment and controls were within the reference ranges in healthy individuals [32, 33]. The normal ranges obtained in diabetes patients could be a reflection of their adequate glycaemic control, although Sharpe et al. [12] reported glycaemic control is not related to mean platelet volume. More studies will elucidate this hypothesis. The results obtained in this study could be skewed in favor of the diabetics because of the unmatched age and gender in the diabetics and controls.

## Conclusion

This study revealed a higher mean platelet count for diabetics on treatment than for non diabetics controls while mean platelet volume was lower in cases than controls. However, both parameters in diabetics on treatment were within normal reference ranges of healthy individuals.
